# Arterial Stiffness and Cerebral Small Vessel Disease

**DOI:** 10.3389/fneur.2018.00723

**Published:** 2018-08-28

**Authors:** Fei-Fei Zhai, Yi-Cong Ye, Si-Yu Chen, Fa-Ming Ding, Fei Han, Xing-Lin Yang, Quan Wang, Li-Xin Zhou, Jun Ni, Ming Yao, Ming-Li Li, Zheng-Yu Jin, Li-Ying Cui, Shu-Yang Zhang, Yi-Cheng Zhu

**Affiliations:** ^1^Department of Neurology, Peking Union Medical College Hospital, Chinese Academy of Medical Sciences and Peking Union Medical College, Beijing, China; ^2^Department of Cardiology, Peking Union Medical College Hospital, Chinese Academy of Medical Sciences and Peking Union Medical College, Beijing, China; ^3^Department of Radiology, Peking Union Medical College Hospital, Chinese Academy of Medical Sciences and Peking Union Medical College, Beijing, China

**Keywords:** arterial stiffness, pulse wave velocity, cerebral small vessel disease, lacunes, white matter hyperintensities, microbleeds, dilated perivascular spaces, brain atrophy

## Abstract

**Background and Objective:** Studies on relations between arterial stiffness and full spectrum of radiological features of cerebral small vessel disease (CSVD) are scarce. We aim to investigate the association of arterial stiffness with lacunes, white matter hyperintensities (WMH), microbleeds (CMBs), dilated perivascular spaces (PVS), and brain atrophy in a community-based sample.

**Methods:** A total of 953 participants (55.7 ± 9.4 years) who underwent brachial-ankle pulse wave velocity (baPWV) and brain magnetic resonance imaging were included. Lacunes, CMBs, and PVS were visually rated. Brain structure and WMH were automatically segmented. Brain parenchyma fraction (BPF), a surrogate index of brain atrophy, was calculated as a ratio of brain parenchyma volume to total intracranial volume. Multivariable logistic and linear regressions were used to investigate the associations between baPWV and CSVD. Subsequently, we explored these associations in strata of age.

**Results:** Increased baPWV was associated with severe PVS in white matter (OR, 1.09; 95%CI, 1.01–1.17; *p* = 0.022), larger WMH volume (β, 0.08; 95%CI, 0.04–0.12; *p* < 0.001), lower BPF (β, −0.09; 95%CI, −0.15– −0.03; *p* = 0.007), and marginally associated with strictly lobar CMBs (OR, 1.11; 95%CI, 1.00–1.23; *p* = 0.055), but not with lacunes. WMH volume mediated the relation between baPWV and BPF. In age subgroup analysis, the association of baPWV with PVS in white matter was stronger among those aged <55 years, whereas the association with brain atrophy was more prominent among those aged ≥55 years. Increased baPWV was associated with larger WMH volume in both younger and older individuals.

**Conclusions:** Increased arterial stiffness was associated with most of imaging markers of CSVD, including PVS in white matter, larger WMH volume, strictly lobar CMBs, and brain atrophy, but not lacunes. The mechanisms underlying these associations and their potential clinical significances warrant further investigations.

## Introduction

The arterial system gradually stiffens with aging and cardiovascular risk factors, especially in large elastic arteries (1). The progressive aortic stiffness lead to increased blood flow pulsatility and transmit excessive pulsatile energy into distal microvasculature (2). Brain is more vulnerable for increased upstream pulsatile energy, because it receives high flow throughout the cardiac cycle and has a low vascular resistance (3, 4). Previous population-based and patient-based studies have demonstrated associations of aortic stiffness with white matter hyperintensities (WMH) (2, 5)and silent brain infarct (2, 6), supporting a link between aortic stiffness and cerebral small vessel disease (CSVD).

As cerebral small-vessels are difficult to directly visualize, we predominantly rely on radiological phenotype as surrogate markers of CSVD. Though WMH, lacunes, CMBs, dilated perivascular spaces (PVS), and brain atrophy, are all regarded as imaging features of CSVD, the underling pathophysiological changes are different (7, 8). WMH and lacunes are mainly ischemic lesions related to hypoperfusion, whereas CMB is hemorrhagic lesions (8). The dilation of PVS may be associated with increased extravasation of interstitial fluid resulting from increased permeability of the blood-brain barrier (9), and diminished drainage of interstitial fluid due to obstruction of perivascular spaces (10). The pathological changes of brain atrophy are heterogeneous, resulting from intrinsic small-vessel disease, or the consequence of lacunes and WMH (11). Therefore, the association pattern between arterial stiffness and each marker of CSVD is likely to be different. However, study investigating the association between arterial stiffness and whole spectrum of CSVD MRI markers is scarce, leaving limited understanding of the impacts of large-artery stiffness on different aspects of cerebral microvascular function.

In the present study, we aim to investigate the association between increased arterial stiffness, measured by brachial-ankle pulse wave velocity (baPWV), and full spectrum of radiological features of CSVD, i.e., lacunes, WMH, CMBs, dilated PVS, and brain atrophy, in a community-based sample, and further explore these associations in strata of age. Since previous studies yielded conflicting results on the association between arterial stiffness and microbleeds at different location (5, 12, 13), we further categorized microbleeds into strictly lobar microbleeds and deep or infratentorial microbleeds.

## Materials and methods

### Participants

The present study was a part of ongoing Shunyi study in China, a community-based cohort designed to investigate the risk factors of cardiovascular diseases and age-related diseases. All inhabitants aged 35 years or older living in five villages of Shunyi, a suburb district of Beijing, were invited. A personal letter which included brief description of the study protocol was sent to every eligible inhabitant. From June 2013 to April 2016, a total of 1,787 subjects agreed to participate and accomplished standard baseline assessments, including structured questionnaires, physical examination, and laboratory tests. All participants were invited to take MRI examination. Among those, 464 participates refused or had contradictions of MRI (cardiac peacemaker, coronary and peripheral artery stents, mental foreign body, or claustrophobia), leaving 1,323 brain MRI were finally performed.

Of 1,323 participates who had brain MRI, 1,036 also accomplished baPWV measurement. We further excluded 75 subjects who had stroke history and 8 because of poor MRI imaging qualities. Thus, the final analysis was performed based on 953 subjects (a flow chart in Supplementary Figure 1).

### Measurement of brachial-ankle PWV

Brachial-ankle PWV was measured using an oscillometric method (VP-1000, Colin, Komaki, Japan) in supine position. Detailed explanation of this device as well as its validity and reproducibility have been provided elsewhere (14). In brief, the device simultaneously recorded pulse waves from the brachial and tibial arteries. The distance between each sampling point and the heart was estimated automatically according to the subject's height. The time interval between the brachium and ankle was defined as the time difference between the waveform of brachium and waveform of the ankle. The average baPWV obtained on both sides was used for further analysis. At the same time, the oscillometric cuffs in the arms could measure blood pressure. The mean blood pressure obtained on both sides was used in the analysis.

### Magnetic resonance imaging

MRI acquisition was performed from July 2014 to April 2016 with a single 3-Tesla Siemens Skyra scanner (Siemens, Erlangen, Germany). Three-dimensional T1-weigted images [repetition time (TR) = 2,530 ms, echo time (TE) = 3.43 ms, inversion time (TI) = 1,100 ms, voxel size = 1 × 1 × 1.3 mm^3^], T2-weighted images (TR = 6,000 ms, TE = 125 ms, 5-mm-thick slices with a 1 mm gap, flip angle = 90°), fluid-attenuated inversion recovery (FLAIR, TR = 8,500 ms, TE = 81 ms, 5 mm-thick slices with a 1 mm gap), and susceptibility-weighted images (SWI, TR = 20 ms, TE = 27 ms, 1.5 mm-thick slices) were included in the routine protocol.

All imaging markers of CSVD were defined according to the Standards for Reporting Vascular Changes on Neuroimaging (STRIVE) (11). Briefly, lacunes were defined as focal fluid-filled cavities of 3–15 mm in diameter situated in the basal ganglia or subcortical white matter and rated on 3D T1-weighted images. Severity of dilated PVS in the basal ganglia (PVS-BG) and white matter (PVS-WM) was rated using a previously established 4-level severity score on 3D T1-weighted images (15). Dilated PVS was defined as cerebrospinal fluid-like signal lesions of round, ovoid, or linear with a diameter generally smaller than 3 mm. CMBs were defined as round or ovoid black lesions (signal void) of smaller than 10 mm in size, and at least half of the lesion was surrounded by brain parenchyma on SWI (16). CMBs were categorized into strictly lobar microbleeds and deep or infratentorial microbleeds based on their location.

The gray matter (GM), white matter (WM), and cerebrospinal fluid (CSF) were automatically segmented on 3D T1-weighted images using SPM12 (http://www.fil.ion.ucl.ac.uk/spm/) and CAT12 toolbox (http://www.neuro.uni-jena.de/vbm/). Total intracranial volume (TIV) was computed as the sum of volume of GM, WM, and CSF. Brain parenchymal fraction (BPF), as a surrogate index of brain atrophy, was the ratio of brain tissue volume (GM+WM) to TIV. WMH were automatically segmented by the lesion growth algorithm as implemented in the LST toolbox (http://www.statistical-modeling.de/lst.html) for SPM at κ = 0.15 (17).

Trained physicians who were blinded to all clinical data rated lacunes, PVS, and CMBs independently. The intrarater agreements were assessed on a random sample of 50 individuals with more than 1-month interval between the first and second readings. The results of kappa for intrarater were as follows: 0.95 for presence of lacunes, 0.71 for PVS-BG scores, 0.61 for PVS-WM scores, and 0.90 for presence of microbleeds.

### Covariates

Diabetes mellitus was defined as self-reported diabetes, or use of oral antidiabetic drugs or insulin, or fasting serum glucose ≥7.0 mmol/L. Smoking status was classified as current smoker (at least within past 1 month) and non-current smoker. Venous blood samples, routinely drawn after an overnight fast, were analyzed for plasma total cholesterol, high-density lipoprotein cholesterol, and plasma glucose levels.

### Statistical analysis

Continuous variables are presented as mean (standard deviation, SD) and categorical variables are presented as frequency (percentage). Degrees of PVS in BG and WM were dichotomized into severe (degree 3 and degree 4) and mild (degree 1 and degree 2). WMH volume was natural logarithmically transformed to normalize the skewness.

Logistic regression and linear regression models were used to investigate the association of baPWV with categorical outcomes (lacunes, CMBs, and PVS) and continuous outcomes (WMH volume and BPF), respectively. All analyses were initially adjusted for age, sex and systolic blood pressure (model 1) and then additionally adjusted for antihypertensive medication, diabetes mellitus, total cholesterol, high-density lipoprotein cholesterol, lipid-lowering medication, and current smoking (model 2). Given nonlinear and exponential increase in aortic PWV with advancing age, especially after the six decades (18–20), we subsequently stratified subjects into younger group (age < 55 years) and older group (age ≥ 55 years) based on the median age. We explored the effect modification of age on the relations between arterial stiffness and CSVD (interaction term age × baPWV) and then we repeated logistic and linear regressions in each subgroup. Given lacunes and WMH were associated with brain atrophy (18), the Sobel test of mediation was used to determine whether lacunes or WMH mediated the directed relations between baPWV and BPF. Results for categorical outcomes are presented as odds ratio (95% confidential interval), and results for continuous outcomes are presented as effect size (β) (95% confidential interval) for per unit increase in baPWV. For all analysis, *p* < 0.05 was considered significant. All analyses were performed using SAS 9.4 (SAS Institute, Cary, NC).

## Results

A total of 953 participants were included. Compared with participants who were excluded, those included in the final analysis were more often female and less often current smoker. Other baseline characteristics were not significantly different between the two groups. Table 1 shows the demographic and clinical characteristics of the present study. The mean age of the study sample was 55.7 years (SD 9.4) and 358 (37.6%) participants were males. The median time interval between pulse wave velocity measurement and MRI was 4 months (interquartile range 1.6–7.9 months).

**Table 1:** Basic characteristics of the study sample.

	**Overall (*n* = 953)**	**Age < 55 (*n* = 461)**	**Age ≥ 55 (*n* = 492)**
**DEMOGRAPHIC AND CLINICAL CHARACTERISTICS**			
Age, y	55.7 (9.4)	47.8 (4.7)	63.2 (6.1)
Male	357 (37.5%)	165 (35.8%)	192 (39.0%)
Systolic blood pressure, mmHg	132.7 (18.7)	128.3 (16.8)	136.8 (19.4)
Diabetes mellitus	153 (16.1%)	47 (10.2%)	106 (21.5%)
Current smoker	218 (23.4%)	101 (22.5%)	117 (24.2%)
Total cholesterol, mmol/L	4.9 (0.9)	4.8 (0.9)	5.0 (1.0)
HDL-cholesterol, mmol/L	1.3 (0.3)	1.3 (0.3)	1.3 (0.3)
Lipid-lowering medication	24 (2.6%)	7 (1.6%)	17 (3.5%)
baPWV, m/s	15.6 (3.2)	14.2 (2.4)	16.9 (3.3)
**MRI MEASUREMENTS**			
Lacunes	144 (15.1%)	26 (5.6%)	118 (24.0%)
Cerebral microbleeds	98 (10.3%)	21 (4.6%)	77 (15.7%)
Strictly lobar microbleeds	48 (5.0%)	12 (2.6%)	36 (7.3%)
Deep or infratentorial microbleeds	50 (5.2%)	9 (2.0%)	41 (8.3%)
Severe PVS-BG (degree 3 and 4)	122 (12.8%)	34 (7.4%)	88 (17.9%)
Severe PVS-WM (degree 3 and 4)	142 (14.9%)	52 (11.3%)	90 (18.3%)
WMH volume, ml	0.9 (0.3, 2.9)	0.4 (0.1, 1.0)	2.4 (0.8, 5.0)
Brain parenchymal fraction, %	76.4 (3.1)	78.2 (2.1)	74.7 (3.0)
Gray matter fraction, %	41.7 (2.1)	42.7 (1.8)	40.7 (1.9)
White matter fraction, %	34.7 (1.9)	35.4 (1.5)	34.0 (2.0)
Total intracranial volume, ml	1403.8 (122.9)	1409.2 (122.5)	1398.7 (123.3)

Values represent mean (SD) for continuous variables and number (%) for categorical variables.

*HDL indicates high-density lipoprotein; baPWV, brachial-ankle pulse wave velocity; PVS-BG, dilated perivascular space in basal ganglia; PVS-WM, dilated perivascular space in white matter, WMH, white matter hyperintensities*.

### Increased baPWV and imaging markers of CVSD

As is shown in Table 2, increased baPWV was associated with severe PVS-WM (OR, 1.09; 95%CI, 1.01–1.17; *p* = 0.022), larger WMH volume (β, 0.08; 95%CI, 0.04–0.12; *p* < 0.001), and lower BPF (β, −0.09; 95%CI,−0.15– −0.03; *p* = 0.007) in fully adjusted models. Although baPWV was not associated with the presence of CMBs, when CMBs were categorized into strictly lobar CMBs and deep or infratentorial CMBs based on their locations, increased baPWV was marginally associated with strictly lobar CMBs (OR, 1.11; 95%CI, 1.00–1.23; *p* = 0.055), but not with deep or infratentorial CMBs (Table 3). Analyses did not reveal significant associations between baPWV and lacunes.

**Table 2:** Association between baPWV and MRI markers of cerebral small vessel disease.

	**Lacunes**		**Microbleeds**		**Severe PVS – WM**		**Severe PVS – BG**		WMH volume*		**BPF**	
	**OR (95%CI)**	***p***	**OR (95%CI)**	***p***	**OR (95%CI)**	***p***	**OR (95%CI)**	***p***	**β (95%CI)**	***p***	**β (95%CI)**	***p***
baPWV, Model 1	1.06 (0.99, 1.14)	0.077	1.03 (0.95, 1.11)	0.459	1.07 (1.00, 1.14)	0.053	0.99 (0.92, 1.07)	0.827	0.09 (0.05, 0.13)	<0.001	−0.10 (−0.16, −0.04)	0.002
baPWV, Model 2	1.03 (0.96, 1.11)	0.377	1.02 (0.94, 1.11)	0.567	1.09 (1.01, 1.17)	0.022	1.00 (0.93,1.09)	0.963	0.08 (0.04,0.12)	<.001	−0.09 (−0.15, −0.03)	0.007

* WMH volume is natural logarithmically transformed.

BaPWV indicates brachial-ankle pulse wave velocity; PVS-WM, dilated perivascular space in white matter; PVS-BG, dilated perivascular space in basal ganglia; WMH, white matter hyperintensities; BPF, brain parenchyma fraction.

*Values represent odds ratio (95% confidential interval) or regression coefficient (β) (95% confidential interval) per 1 m/s increase in baPWV. Model 1: adjusted for age, sex, and systolic blood pressure. Model 2: adjusted for age, sex, systolic blood pressure, antihypertensive medication, diabetes mellitus, total cholesterol, high-density lipoprotein cholesterol, lipid-lowering medication, and current smoking*.

**Table 3:** Association between baPWV and microbleeds based on the location.

	**Strictly lobar CMBs**		**Deep or infratentorial CMBs**	
	**OR (95%CI)**	***p***	**OR (95%CI)**	***p***
baPWV, Model 1	1.10 (1.00, 1.21)	0.050	0.95 (0.85, 1.07)	0.394
baPWV, Model 2	1.11 (1.00, 1.23)	0.055	0.95 (0.84, 1.06)	0.359

BaPWV indicates brachial-ankle pulse wave velocity; CMBs, cerebral microbleeds.

*Model 1: adjusted for age, sex, and systolic blood pressure. Model 2: adjusted for age, sex, systolic blood pressure, antihypertensive medication, diabetes mellitus, total cholesterol, high-density lipoprotein cholesterol, lipid-lowering medication, and current smoking*.

Additionally, we employed the Sobel test to determine whether WMH volume or the presence of lacunes mediated the directed relation between baPWV and BPF. The Sobel test showed that WMH volume did mediate the relation between baPWV and BPF (*z*-value = 3.74, *p* < 0.001), whereas lacunes did not mediated the relation between baPWV and BPF (*z*-value = 1.34, *p* = 0.18). After adjusting for WMH, the direct relation between baPWV and BPF became non-significant (β, −0.03; 95%CI,−0.09–0.02; *p* = 0.24).

### Increased baPWV and imaging markers of CSVD in age subgroups

We observed effect modification by age in the associations of baPWV with severe PVS-W (*p* = 0.030 for interaction) and lower BPF (*p* = 0.029 for interaction). The interaction terms in other models were not significant. When we stratified the participants into two age subgroups according to the median (55 years), the effect size in the association of baPWV with PVS-WM was greater in the younger group than in the older group (OR = 1.21, *p* = 0.007 vs. OR = 1.05, *p* = 0.238). By contrast, the association between baPWV and BPF was only significant in older group (β, −0.08; 95%CI, −0.16–0.00; *p* = 0.042). However, this association attenuated in fully adjusted model (Table 4).

**Table 4:** The association between baPWV and MRI markers of cerebral small vessel disease stratified by age.

	**Lacunes**		**Microbleeds**		**Severe PVS – WM**		**Severe PVS – BG**		WMH volume*		**BPF**	
	**OR (95%CI)**	***p***	**OR (95%CI)**	***p***	**OR (95%CI)**	***p***	**OR (95%CI)**	***p***	**β (95%CI)**	***p***	**β (95%CI)**	***p***
Age < 55 (*n* = 461)												
baPWV, Model 1	1.06 (0.91, 1.24)	0.468	0.99 (0.82, 1.21)	0.954	1.15 (1.02, 1.31)	0.023	1.10 (0.96, 1.27)	0.179	0.12 (0.05, 0.19)	0.001	−0.09 (-0.20, 0.01)	0.070
baPWV, Model 2	0.96 (0.80, 1.16)	0.688	1.00 (0.82, 1.22)	0.993	1.21 (1.05, 1.38)	0.007	1.19 (1.00, 1.40)	0.045	0.11 (0.04, 0.19)	0.003	−0.08 (-0.18, 0.02)	0.115
Age ≥ 55 (*n* = 492)												
baPWV, Model 1	1.08 (1.00, 1.16)	0.064	1.04 (0.95, 1.13)	0.399	1.04 (0.96, 1.13)	0.324	0.96 (0.88, 1.05)	0.403	0.07 (0.03, 0.12)	< 0.001	−0.08 (-0.16, 0.00)	0.042
baPWV, Model 2	1.06 (0.98, 1.15)	0.171	1.03 (0.94, 1.13)	0.498	1.05 (0.97, 1.15)	0.238	0.97 (0.88, 1.06)	0.495	0.06 (0.02, 0.11)	0.009	−0.05 (−0.13, 0.03)	0.219

BaPWV indicates brachial-ankle pulse wave velocity; PVS-WM, dilated perivascular space in white matter; PVS-BG, dilated perivascular space in basal ganglia; WMH, white matter hyperintensities; BPF, brain parenchyma fraction.

Values represent odds ratio (95% confidential interval) or regression coefficient (β) (95% confidential interval) per 1 m/s increase in baPWV. Model 1: adjusted for age, sex, and systolic blood pressure. Model 2: adjusted for age, sex, systolic blood pressure, antihypertensive medication, diabetes mellitus, total cholesterol, high-density lipoprotein cholesterol, lipid-lowering medication, and current smoking.

* *WMH volume is natural logarithmically transformed*.

Increased baPWV was associated with larger WMH volume in both participants younger than 55 years and participants aged 55 years and older, and this association was not modified by age (*p* = 0.258 for interaction). That is, increased baPWV related to severe WMH in both younger and older individuals and the effect size of these relations was not significantly different.

## Discussion

This study assessed the associations of arterial stiffness and imaging markers of CSVD in a community-based sample. We found that arterial stiffness was associated with severe PVS-WM, larger WMH volume, strictly lobar CMBs, and brain atrophy. The association of baPWV and brain atrophy was mediated by WMH. In subgroup analysis, the association of baPWV with PVS-WM was stronger among those younger than 55 years, whereas the association with brain atrophy was more prominent among those aged 55 years and older. Increased baPWV was associated with larger WMH volume in both younger and older individuals.

Our findings that arterial stiffness was associated with larger WMH volume and brain atrophy were consistent with previous studies (6, 19–21). In subgroup analysis, arterial stiffness was associated with larger WMH volume in both younger and older individuals, indicating that arterial stiffness-related white matter injury may occur even in young adults. This finding has been supported by recent works in the Framingham study (20). We also found that the association of arterial stiffness and brain atrophy was mediated by WMH. There are several plausible explanations for the mediation effect of WMH on the relation between baPWV and brain atrophy. It is conceivable that stiffness-related WMH contributes to volumetric loss of white matter constituents such as myelin, axons, and oligodendrocyte (22). An alternative possibility is that a “disconnection phenomenon” of cortical region due to white matter denervation lead to gray matter atrophy (22).

The relations of arterial stiffness and dilated perivascular spaces, another CSVD marker, has not been described. In the present study, we found that increased baPWV was associated with severe PVS-WM. Possible explanation is that, with the progression of aortic stiffness, more mechanical pulsatile energy is transmitted into peripheral microvascular beds. The pulsatile energy could impair capillary endothelium, increase the permeability of blood-brain barrier, and ultimately lead to greater extravasation of fluid into perivascular space (9) as well as reduced interstitial fluid drainage (10), which manifest as dilated PVS on MRI. Interestingly, we found that the association between increased baPWV and severe PVS was prominent in the younger group. Possible explanation is that, in individuals under 55 years who were exposed to fewer vascular risk factors, arterial stiffness can explain certain variance of PVS enlargement, while this effect might be attenuated with aging as multiple risk factors accumulated. What's more, PVS dilation may represent an epiphenomenon of early stage of arteriolar wall dysfunction, which may subsequently contribute to perivascular parenchyma damage, such as white matter hyperintensities. Further studies are required to replicate and substantiate our findings.

Previous studies yielded conflicting results on the association between arterial stiffness and microbleeds. Ochi et al. (12) found an association between arterial stiffness and presence of CMBs irrespective of their location in apparently healthy individuals. Poels et al. (5) found arterial stiffness was associated with deep or infratentorial CMBs in individuals with uncontrolled hypertension, while Henskens et al. (13) failed to find such association in hypertensive patients. In the present study, we found an association between baPWV and strictly lobar CMBs, a kind of microbleeds resulting from cerebral amyloid angiopathy. Underlying mechanisms are still unknown. Previous findings (23, 24) that baPWV related to both baseline β-amyloid burden and β-amyloid progression on Aβ-PET in non-demented elderly adults may provide possible explanation for our results. Hughes's recent findings (21) that central arterial stiffness was associated with microbleeds and CSVD were also consistent with our results.

The AGES-Reykjavik study (2) (mean age, 75–76 years) and the Framingham study (6) (mean age, 61 years) have shown a higher prevalence of silent brain infarcts in persons with higher arterial stiffness values, but such association was not observed in our study. These discrepancies may be primarily attributable to our sample's relatively younger age (mean age, 55.7 years), hence a lower prevalence of lacunar infarcts.

Our study has the advantage of the population-based design and a large sample. There are still several limitations. First, cross-sectional, observational study design limits our ability to establish causal relationships between arterial stiffness and CSVD. Second, although previous studies have revealed the validity of baPWV as an index of aortic stiffness (14, 25), direct assessment using carotid-femoral pulse wave velocity, a gold standard for aortic stiffness assessment, on certain size of the patient sample will be a good comparison with current study. Third, because of relatively low prevalence of microbleeds, there is a risk of overfitting in regression models. Forth, as subgroup analysis was conducted in the study, we should be cautious with the subgroup differences and avoid over-interpretation of the results because of the multiple comparison, even though we had performed a formal statistical test for interaction between baPWV and age before subgroup analysis.

Our results expend previous studies and provide further evidences of the association between arterial stiffness and CSVD. Findings of arterial stiffness correlates of CSVD can potentially help physicians to identify populations at high risk of CSVD, and treatment on arterial stiffness may be helpful to prevent and control the progression of CSVD.

## Ethics statement

This study was carried out in accordance with the recommendations of the Medical Review Ethics Committee of Peking Union Medical College Hospital. All subjects gave written informed consent in accordance with the Declaration of Helsinki. The protocol was approved by the Medical Review Ethics Committee of Peking Union Medical College Hospital (reference number: B-160).

## Author contributions

F-FZ drafted the manuscript, conducted the statistical analysis, and interpreted the data. S-YC, FH, QW, and M-LL contributed to brain imaging analysis. Y-CY, F-MD, and X-LY acquired baPWV data and provided expertise on baPWV analysis. L-XZ, JN, MY, L-YC, Z-YJ, and S-YZ conceived and designed the study. Y-CZ was the principal investigator of the study and was responsible for the study conception and interpretation of data and had final responsibility for the decision to submit for publication.

### Conflict of interest statement

The authors declare that the research was conducted in the absence of any commercial or financial relationships that could be construed as a potential conflict of interest.
