# Supplementary material for: Arterial Stiffness and Cerebral Small Vessel Disease
Source: Front Neurol. 2018 Aug 28;9:723. doi: 10.3389/fneur.2018.00723 (PMC6121106; doi:10.3389/fneur.2018.00723)
Supplement: Supplementary file 1 [file Data_Sheet_1.docx]

**Supplementary materials**


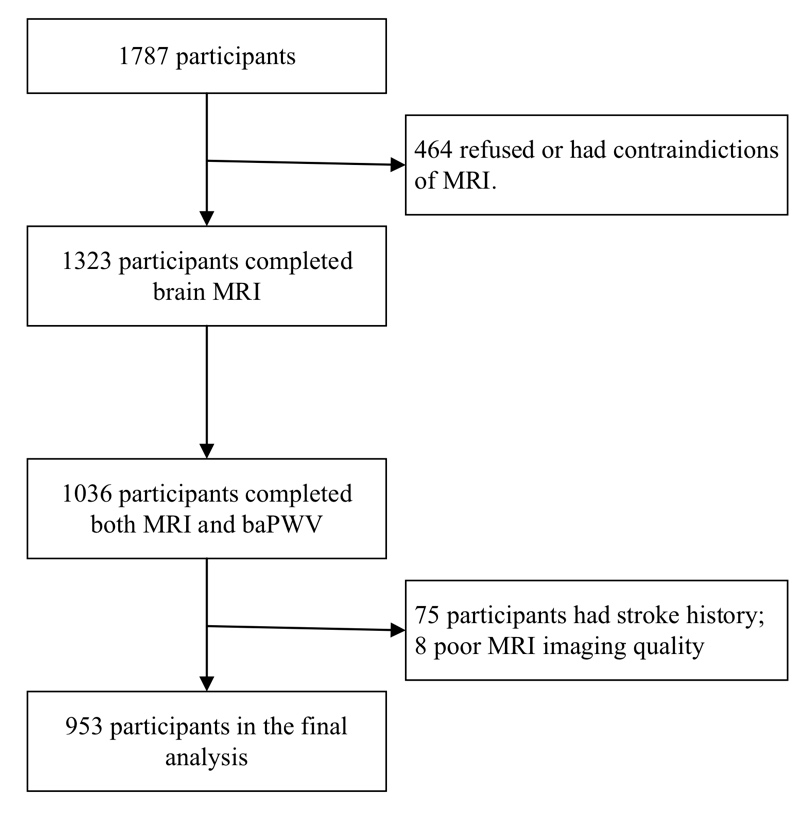


**Supplementary figure 1**. Flow chart of participants inclusion and exclusion in the study. MRI = magnetic resonance imaging; baPWV = brachial-ankle pulse wave velocity
